# Shoulder muscle forces during driving: Sudden steering can load the rotator cuff beyond its repair limit

**DOI:** 10.1016/j.clinbiomech.2015.06.004

**Published:** 2015-10

**Authors:** Petros Pandis, Joe A.I. Prinold, Anthony M.J. Bull

**Affiliations:** Department of Bioengineering, Imperial College London, South Kensington Campus, London SW7 2AZ, UK

**Keywords:** Musculoskeletal biomechanics modelling, Shoulder functionality, Upper limb muscle forces, Driving, Steering, Daily activities, Supraspinatus, Surgical repair

## Abstract

**Background:**

Driving is one of the most common everyday tasks and the rotator cuff muscles are the primary shoulder stabilisers. Muscle forces during driving are not currently known, yet knowledge of these would influence important clinical advice such as return to activities after surgery. The aim of this study is to quantify shoulder and rotator cuff muscle forces during driving in different postures.

**Methods:**

A musculoskeletal modelling approach is taken, using a modified driving simulator in combination with an upper limb musculoskeletal model (UK National Shoulder Model). Motion data and external force vectors were model inputs and upper limb muscle and joint forces were the outputs.

**Findings:**

Comparisons of the predicted glenohumeral joint forces were compared to *in vivo* literature values, with good agreement demonstrated (61 SD 8% body weight mean peak compared to 60 SD 1% body weight mean peak). High muscle activation was predicted in the rotator cuff muscles; particularly supraspinatus (mean 55% of the maximum and up to 164 SD 27 N). This level of loading is up to 72% of mean failure strength for supraspinatus repairs, and could therefore be dangerous for some cases. Statistically significant and large differences are shown to exist in the joint and muscle forces for different driving positions as well as steering with one or both hands (up to 46% body weight glenohumeral joint force).

**Interpretation:**

These conclusions should be a key consideration in rehabilitating the shoulder after surgery, preventing specific upper limb injuries and predicting return to driving recommendations.

## Highlights

• Physical experiments and musculoskeletal modeling are used to quantify shoulder muscle forces during steering.
• Driving position on its own significantly affects muscles forces.
• Under certain conditions, supraspinatus muscle forces during sudden steering are close to the failure strength of supraspinatus repairs.

## 1 Introduction

Driving is one of the most common everyday tasks. The function of the shoulder in driving is to provide actuation to steering and thus injuries to this structure can not only inhibit function, but these injuries might also be exacerbated by the steering function. The shoulder is a joint for which the active stabilisers (rotator cuff muscles) provide a greater proportion of restraint than the articulating surfaces or ligaments when compared to joints such as the hip or knee (Kedgley et al., 2008; Veeger and van der Helm, 2007; Yanagawa et al., 2008). Therefore, in order to understand the function of the shoulder, it is important to identify muscle activation levels.

Prior work using surface electromyography (EMG) has shown low correlation between the activity of the shoulder muscles and the movement of the steering wheel, and therefore, little is known about which muscles are active during driving (Solveig and Johnsson, 1975). Due to the fact that surface EMG is normally used for large superficial muscles (such as deltoid, trapezius, biceps and triceps), not all the muscles can be assessed simultaneously. According to the results of Solveig and Johnsson (1975), both the main flexors and extensors of the elbow are not the prime movers in turning the wheel. However, prior work has not allowed the quantification of the activation of deep muscles, such as the rotator cuff muscles, and the effect of driver position on this activation.

Therefore, the aim of this study was to quantify rotator cuff muscle forces during sudden steering in different postures. Due to ethical and practical limitations with needle EMG, a combined musculoskeletal modelling with kinematic and kinetic measurement approach is taken.

## 2 Materials and methods

### 2.1 Subjects

Eight healthy right-hand male subjects with no history of shoulder pathology participated in the study (age: 25 SD 4 years, height: 178 SD 10 cm, body mass: 71 SD 12 kg). Informed consent was obtained from each subject, and ethical approval was granted from Imperial College Research Ethics Committee (ICREC_12_1_15).

### 2.2 Apparatus

**Fig. 1:**
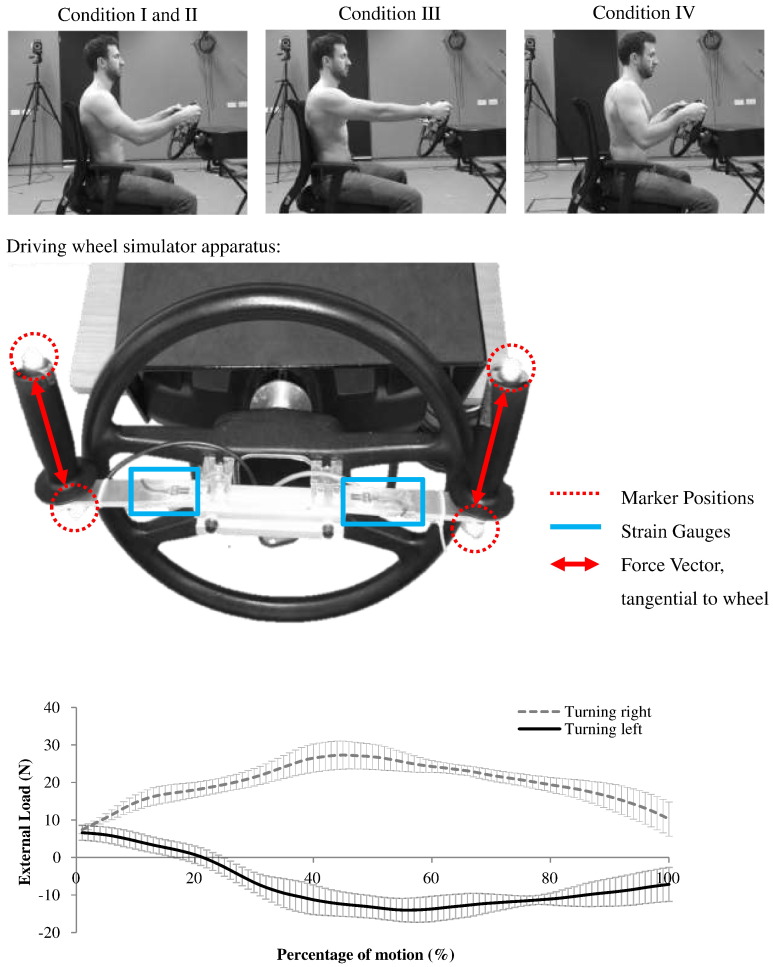
(Top) Subject demonstrating three driving positions. (Middle) Modified driving wheel simulator showing marker positions with resultant force vectors and strain gauges. (Bottom) Mean external loading on the right hand and intersubject values of SD during steering right and left in a comfortable driving position (Condition I).

A driving simulator was designed and built with a user interface that instructs the subject to suddenly turn right or left in a random order; simulating an avoidance task. The simulator was set up to assess right upper limb function during turning to the left or right. The initial design of the simulator was conducted by Haynes (2005) to simulate driving a standard family vehicle. In this study, the simulator was modified to measure the external force. The task is completed when the wheel has been turned 65°. The forces at the hands are measured by a calibrated, strain-gauge-instrumented attachment on the driving rig (Fig. 1). Specifically, the system consists of four TML (FLA-5-23) strain gauges (Tokyo Sokki Kenkyujo Co. LTD, Tokyo) at each handle, using a full Wheatstone bridge configuration with a bending strain gauge arrangement. Torque resistance on the wheel is set at 4 Nm in order to simulate a standard driving torque (Li and Xian, 2013). Motion of the subject and simulator wheel is tracked using optical motion tracking (VICON Motion Tracking System, VICON, Oxford, UK) acquiring data at 100 Hz (Fig. 1). A micro analogue 2 (FE-366-TA) amplifier (Flyde Electronic Laboratories LTD, Preston) was used for the communication between the strange gauges and the VICON capture system. The output voltage was given in the VICON system synchronised with the motion tracking data. Four markers were placed on the handles, two on each handle (Fig. 1) to define the external force vector's direction for input into the model.

### 2.3 Protocol

Optical motion tracking markers were placed on the subject according to the landmarks recommended by the ISB (Wu et al., 2005) considering the upper limb as 5 segments: thorax, scapula, humerus, radius, and ulna. Specifically, the markers were placed on the radial styloid (most cauda-lateral point), ulna styloid (most cauda-lateral point), right and left acromioclavicular joint (most dorsal point), incisura jugularis (suprasternal notch), processus xiphoideus (most caudal point on the sternum), processus spinosus (on the 7th cervical vertebra) and processus spinosus (on the 8th thoracic vertebra). The position of the medial and lateral epicondyle was defined according to the position of the technical coordinate system of a pointer's triad in static trials before data collection. During motion capture, the position of each epicondyle was reconstructed through geometric calculations with respect to an upper arm technical coordinate frame (Cappozzo et al., 1995). In order to describe the orientation of the upper limb joints in the 3D Euclidean space, the shoulder, elbow and scapular rotations were calculated using Euler angles with z-x′-y″ Cardan sequence. For the elbow joint, the rotations about z, y and x axes are the elbow flexion/extension, pronation/supination and varus/valgus (tilt), respectively, and for the glenohumeral joint, the rotations about z, y and x axes are forward flexion/extension, external/internal rotation and abduction/adduction, respectively (Wu et al., 2005).

Subjects were asked to position themselves on an adjustable seat in their ‘comfortable driving position’. In this position, they were instructed to respond to the randomised instruction from the simulator to turn left or right at maximum speed. Six left and six right turn recordings were taken. Each subject repeated the measures for the following conditions (Fig. 1):

I Comfortable seated position, both hands on wheel.
II Comfortable seated position, single hand on wheel.
III Distant seated position, both hands on wheel.
IV Close seated position, both hands on wheel.

These positions were chosen to allow comparison with literature data (Westerhoff et al., 2009) and analysis of the full range of driving positions that have been described subjectively as comfortable for driving (Park et al., 2000; Rebiffe, 1966).

### 2.4 Modelling and analysis

The motion data and the external force at the hand (Fig. 1) were used as inputs into the UK National Shoulder Model (UKNSM; Charlton and Johnson, 2006), which was used to model upper limb muscle forces in the right shoulder. The model is an inverse dynamics musculoskeletal model of the upper limb including 91 muscle elements crossing five joints (sternoclavicular, acromioclavicular, scapulothoracic, glenohumeral and elbow). Model verification is performed through comparison to EMG and instrumented-implant-validated model results (Charlton and Johnson, 2006; Prinold et al., 2013). The load-sharing optimisation minimises the sum of the squared muscle stresses, a criterion that results in low amounts of co-contraction. The wrist is considered as a fixed joint and thus the load-sharing optimisation is not applied to this joint. The tangential force vector defined on the steering wheel is applied through the centre of mass of the hand and translated to the wrist position. Subject-specific measurements, such as upper limb segmental lengths, and body weight and height are used for scaling the musculoskeletal model body segment parameters. Muscle physiological cross-sectional areas and maximum muscle stress (100 Ncm^− 2^) were not scaled. Scapula kinematics are derived from regression equations based on the humero-thoracic position (Charlton and Johnson, 2006). The model outputs were glenohumeral joint reaction force, muscle forces and muscle activation (maximum predicted muscle force in the driving task divided by the muscle's maximum producible force; Table 1). Rotator cuff muscles and selected other muscles, qualitatively observed from the model results to be most active, were analysed (Table 1).

SPSS Version 21 (IBM Corporation, New York, USA) was used for the statistical analysis. Differences between the four loading states were assessed using a repeated measures one-way ANOVA with pairwise-comparison analysis using the Holm–Bonferroni method. ‘Condition I' was used as the reference value in order to identify the differences between the different conditions.

## 3 Results

**Fig. 2:**
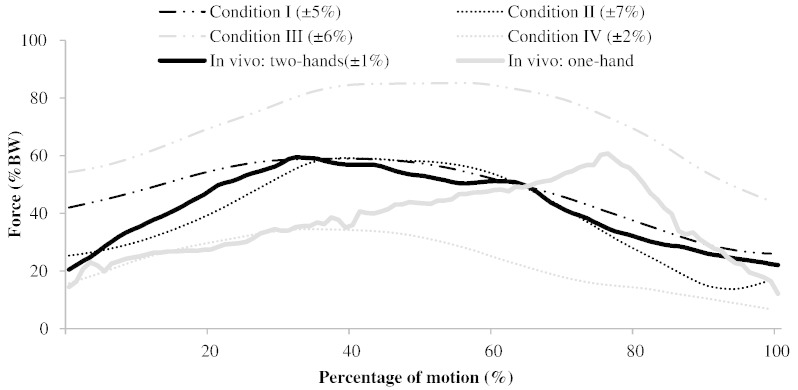
Mean values of the GH joint contact force during four driving conditions compared to *in vivo* data from instrumented GH implants. 0% indicates that the wheel is turned 0°; just beginning to turn, while 100% indicates the wheel is turned 65°. The average time representing the 100% of the motion is 0.268 SD 0.065 sec. The mean *in vivo* measurements for turning right (or left in the case of the left-handed subject) are also presented (Westerhoff et al., 2009). Note that the one-handed *in vivo* wheel turn is the data from a single subject because it is the only subject's data that are considered complete (OrthoLoad, 2014). The mean inter-subject standard deviations are shown in the insert.

The mean maximum glenohumeral joint force with the subjects sitting in a comfortable position turning right was found to be 61.1 SD 7.8% BW (≈ 425 N and where BW refers to body weight; Fig. 2) compared to 39.4 SD 6.0%BW turning left. Higher muscle forces and activations were found turning right, therefore this is considered to have greater clinical importance and only these forces are presented. The pattern of the glenohumeral loading throughout the motion is presented and compared to *in vivo* measurements (Fig. 2). The difference in muscle and joint forces that are found in this study are a result of both different kinematics (Fig. 5) during steering as well as the external forces at the hand (Fig. 1).

**Fig. 3:**
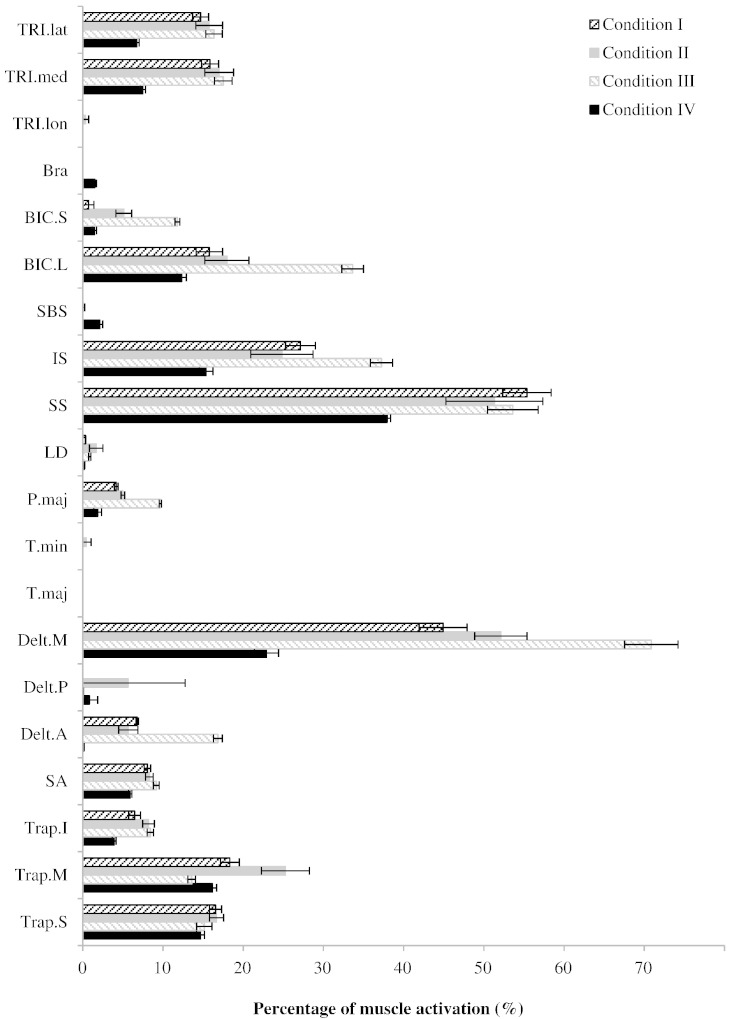
Mean values (error bars: inter-subject standard deviation) of the maximum muscle activation during steering right in the four driving positions. Refer to Table 1 for the abbreviation/muscle name.

The ‘distant to the wheel’ driving position (Condition III) led to the highest individual muscle activation (71 SD 3% in the medial deltoid head) and also generally led to higher activations in the other muscles (Fig. 3). Conversely, driving close to the wheel (Condition IV) significantly reduced the muscle forces (p < 0.05; Table 2) and reduced the glenohumeral joint force 61.1 SD 7.8% BW to 35.3SD 3.1 %BW (p < 0.05). The largest difference between the two different driving positions is observed in the medial deltoid head. The maximum muscle activation in the comfortable position is similar between driving with one hand and two hands (Fig. 3), with significant differences found in some cases (Table 2).

**Fig. 4:**
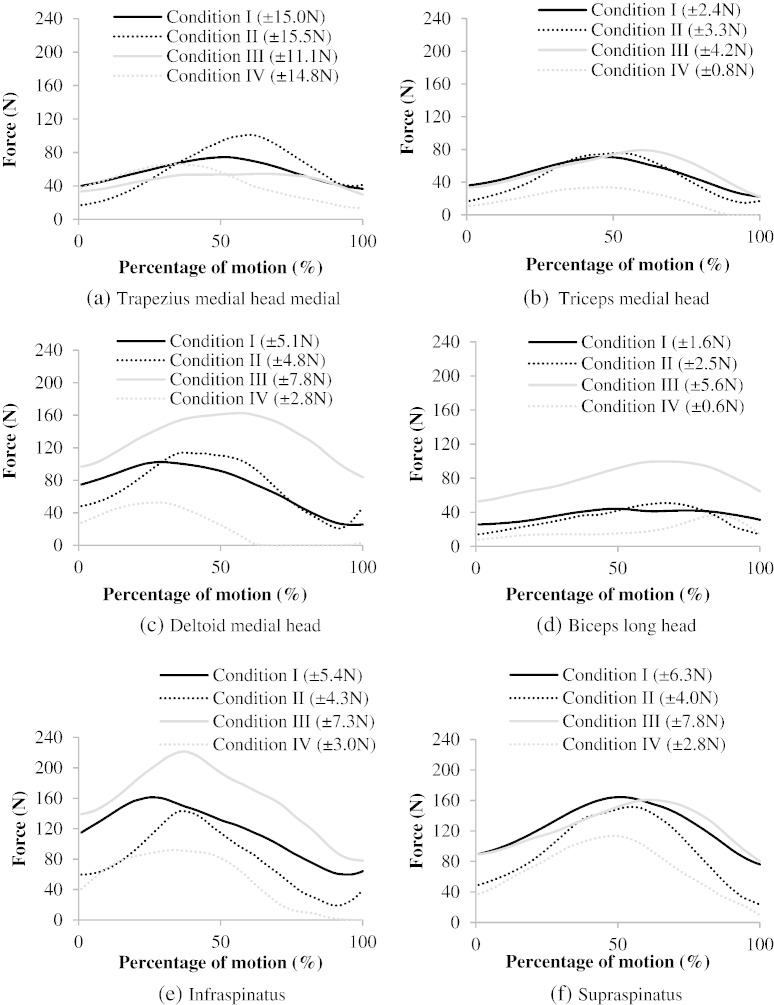
Mean values of: (a) trapezius medial head, (b) triceps medial head, (c) deltoid medial head, (d) biceps long head, (e) infraspinatus, (f) supraspinatus muscle forces in the four different driving conditions. 0% indicates that the wheel is turned 0°; just beginning to turn, while 100% indicates the wheel is turned 65°. The average time representing the 100% of the motion is 0.268 SD 0.065 sec. Mean inter-subject standard deviations are shown in the graph's insert.

The six most active muscles: trapezius medial head, triceps medial head, deltoid medial head, supraspinatus, infraspinatus, and long head of biceps are presented during right steering in the four different driving positions (Fig. 4). This facilitates understanding of each muscle's role during the motion.

## 4 Discussion

This study presents musculoskeletal model predictions of muscle and joint loads during driving - an important daily activity. The predicted GH joint reaction forces are comparable to the literature values for *in vivo* GH joint reaction forces (Fig. 2). The predicted muscle actions are also explicable; the middle deltoid is the most active muscle in maintaining the arm in a raised position, using its strong extension moment arm (Figs. 3 and 4; Ackland et al., 2008); the supraspinatus and long head of biceps also support the weight of the arm (Fig. 3) while applying a well-directed line of action for centralising the humeral head on the glenoid (Ackland and Pandy, 2009); the trapezius muscles are active in maintaining the elevation of the shoulder girdle, as observed in similar positions, such as desk-work (Rasmussen and De Zee, 2010); infraspinatus and short head of triceps then act to actuate the steering task, flexing the shoulder and extending the arm, respectively.

**Fig. 5:**
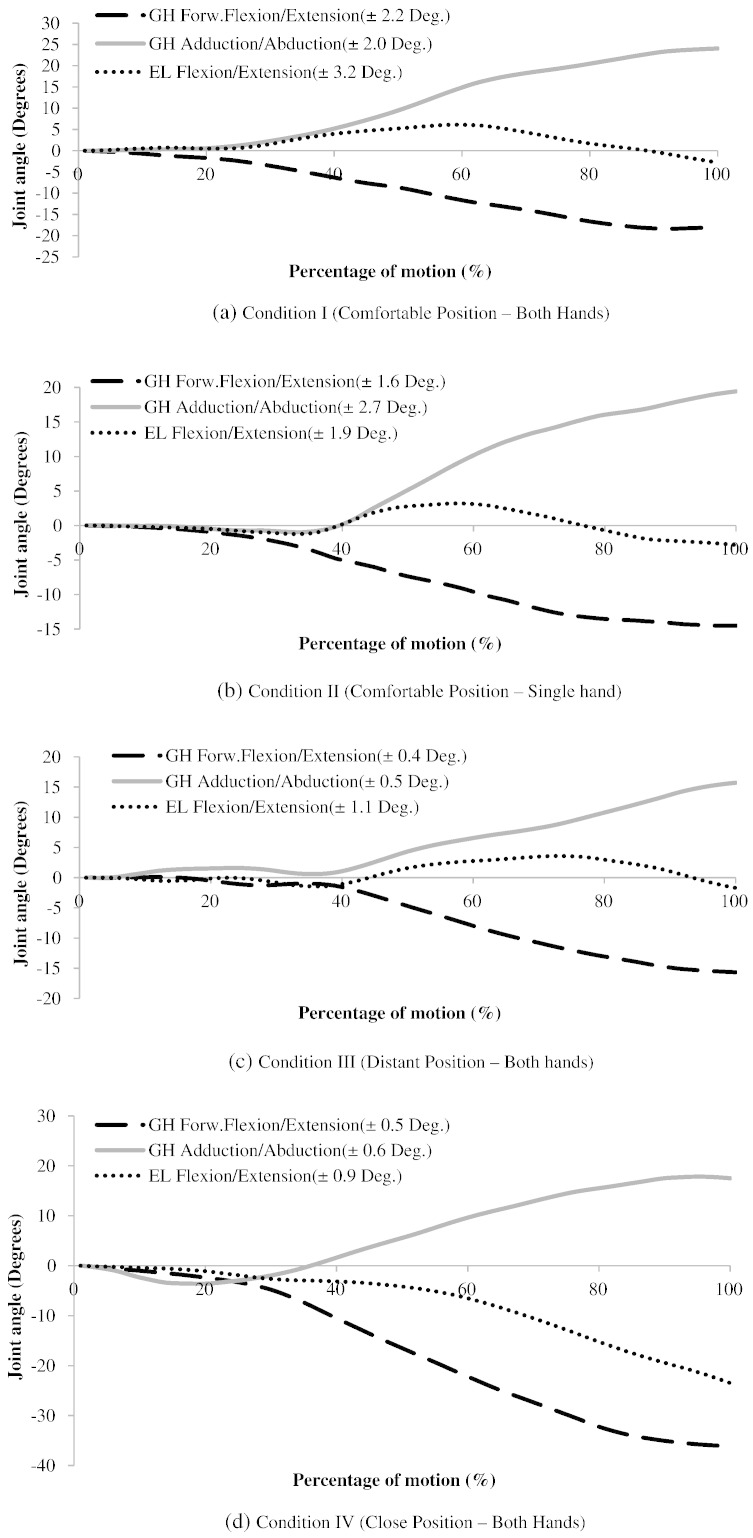
Mean GH and EL joint angles for steering right for all four conditions. The mean inter-subject SD values are not shown as error bars for clarity of presentation. Mean SD values are shown in the graph's insert.

The modelling limitations of this study are similar to those of current generic musculoskeletal models. Scapula kinematics was derived from regression equations rather than measured kinematics. However, given the small range of motion at the GH joint (< 24° flexion/extension and < 18° abduction/adduction), the effects of this should be relatively insignificant—Fig. 5 shows the kinematics for the GH joint for all driving conditions. The modelling of the wrist is simplified to a fused joint. Although there will be active muscles driving the hand to grip the wheel, it is observed that the muscle forces at the elbow are relatively small (Fig. 3), and therefore, the effect of this simplification should not be significant further up the model chain; which is the focus on this study. Others have found that high levels of grip (up to 50% MVC) do not have a significant effect on shoulder muscle activation at low levels of arm elevation (Palmerud et al., 2000; Sporrong et al., 1996).

The subjects in this study are young adults. Although elderly populations are generally associated with rotator cuff injury (Minigawa and Itoi, 2006), it is known that a history of trauma is the most strongly correlated factor with rotator cuff tears (Yamamoto et al., 2010) and the conclusions relating to the loading of the shoulder muscles and shoulder joint are relevant to the very large population that regularly drive (38 million driving licenses held in the UK; data.gov.uk, 2013), regardless of age.

Westerhoff et al. (2009) measured joint forces *in vivo* for steering with both hands and sitting in a comfortable position by using telemeterised shoulder implants in four patients. One of the subjects performed a motion that was significantly different from the others and the motion performed in this study (OrthoLoad, 2014) and is therefore not included in the presented analysis. In order to improve the similarity between experimental methodologies, only the right turn portion of the motion is considered from the *in vivo* data. The predicted values of the joint forces in Condition I (61.1 SD7.8 %BW mean peak) are similar to the *in vivo* values (59.9 SD1.1 %BW mean peak; Fig. 2). The pattern of the joint load from the telemeterised GH joint implant is also similar to this study; with a peak value found at about 40% of the motion (Fig. 2). The differences between the results of these studies could be explained by: the different torque resistance in the wheel (57% lower here), the different speed at which the task was performed (faster here) and the amount of wheel turn (90° right compared to 65° right here). Shoulder muscle activations are strongly and positively correlated with the steering resistance torque (Pick and Cole, 2006). All the subjects used by Westerhoff et al. (2009) are patients with osteoarthritis of the shoulder, therefore the effects of surgery and learnt coping mechanisms must be considered The comparison with literature should only be considered as an approximate test of magnitude and pattern, because of the high inter-individual variation of joint forces during steering (Westerhoff et al., 2009), small sample size of *in vivo* data, differences in experimental methodology and the fact that the data from the literature are for patients with an endoprosthesis with likely high level of co-contraction to achieve joint stability.

Steering right was found to produce higher joint loads in the right shoulder; the literature is contradictory (Westerhoff et al., 2009), although the mean trends in this result are not described; the fact is simply stated. The discussed differences between the studies may contribute to this difference, particularly the fact that the subjects have replacement shoulder implants. These subjects are therefore likely to have a reduced range of motion (Bryant et al., 2005; Ludewig et al., 2009), meaning that the upward portion of the driving (turning left with the right hand) becomes an activity that is potentially near the edge of their range of motion, particularly at 90° of wheel turn—leading to increased joint forces. One limitation could also be that inverse-dynamics musculoskeletal models are not currently able to predict muscle co-contractions at the GH joint. This is likely to strengthen the discussed conclusions related to high supraspinatus and infraspinatus loading, since these muscles are expected to co-contract (Veeger and van der Helm, 2007).

The results show that most driving conditions caused moderate (> 30%) to high activation (> 50%) of supraspinatus and deltoid with some moderate activation of infraspinatus (Table 2). Repeated high muscle activation could lead to muscle fatigue or even overload; particularly since supraspinatus and deltoid are potentially loaded eccentrically (Lieber and Friden, 1993; Proske and Morgan, 2001). Moreover, these muscles presented nearly two times higher activation than any other muscle of the upper limb; therefore, injury to one of these muscles could lead to a dangerous increase of the activation of the other muscles to compensate. As supraspinatus and deltoid act together, injury or weakness in one of these muscles may mean that the other muscle will be unable to compensate for the load due to the already high activation when both muscles are functioning normally. This may have implications for joint instability, particularly in the case of the supraspinatus.

Driving close to the wheel reduces the forces generated by the supraspinatus muscle by 31% (peak forces) and 45% (mean forces; Fig. 4) and therefore reduces the risks of overloading (including the discussed rotator cuff repair below). This is expected because the moment at the shoulder caused by the mass of the arm will be reduced when compared to the comfortable (Condition I), and particularly the distant (Condition III), driving conditions.

The reduced but similar GH joint loads in driving with one hand (Fig. 2; Condition II) may be explicable because when turning right, it is not necessary to overcome the mass of the other hand and arm, as is the case of driving with both hands (Condition I). The pattern of the GH joint force between one and two hand driving has some similarity to the *in vivo* pattern of loading (Fig. 2; Westerhoff et al., 2009), although these data are only taken from one subject and are thus of limited utility.

Estimates of the cumulative annual incidence of rotator cuff disorders vary from 7% to 25% in the Western general population (Bilal, 2011), while the mean failure strengths for single-row repairs and double-row repairs of supraspinatus are 224 SD 148 N and 325 SD 74 N, respectively Smith et al. (2006). In this study, supraspinatus forces in driving ‘distant to the wheel’ (Condition III) were as high as 164 SD 27 N; 73% of the failure load. As the glenohumeral joint is inherently unstable, co-contraction is often seen with upper limb motions. However, as this computational method predicts only low levels of co-contraction due to the mathematics of the model cost function, so it is likely to underestimate this co-contraction. Therefore, it is expected that in some cases, loading may actually be higher than quantified here; this points to the need for care in a post-operative rotator cuff repair period.

As rotator cuff muscles weaken with age, there would be an expected change in the kinematics of steering. This would result in different model outputs. Therefore, further work should focus on increasing the sample size of the study to allow for variations in gender, age and body dimensions to be assessed. In addition, driving positions could be parameterised in terms of distance to the wheel which could then lead to the definition of a safe driving position that is associated with shoulder pathology. Finally, as with all musculoskeletal modelling studies, further validation could be conducted with EMG and instrumented implants.

## Conflict of interest statement

The authors declare that there are no financial or personal relationships with people or organisations that have inappropriately influenced this work.

## Figures and Tables

**Table 1:** Muscles that are considered in this study and their abbreviations. The number of elements used in the musculoskeletal model to represent each muscle. The maximum allowable muscle force, used as a constraint in the musculoskeletal modelling (Charlton and Johnson, 2006), is also shown.

Muscle	Abbreviation	Number of elements	Max. muscle force (N)
Trapezius S.(Superior fibres)	Trap.S	3	330
Trapezius M.(Middle fibres)	Trap.M	2	410
Trapezius I. (Inferior fibres)	Trap.I	11	560
Serratus anterior	SA	9	1050
Deltoid anterior	Delt.A	2	380
Deltoid posterior	Delt.P	2	620
Deltoid middle	Delt.M	1	230
Teres major	T.maj	1	410
Teres minor	T.min	1	210
Pectoralis major (thorax origin)	P.maj	5	950
Latissimus dorsi	LD	5	660
Supraspinatus	SS	1	300
Infraspinatus	IS	3	600
Subscapularis	SBS	3	780
Biceps long head	BIC.L	1	297
Biceps short head	BIC.S	1	283
Brachialis	Bra	2	524
Triceps long	TRI.long	2	470
Triceps medial	TRI.med	2	452
Triceps lateral	TRI.lat	2	420

**Table 2:** Muscle forces and activation (n = 8) for turning right (N.S.D. = no significant difference, < 0.05 = significant difference at p < 0.05).

Muscle	Condition	Mean minimum force (N)	Mean maximum force (N)	Mean force (N)	Inter- subject SD (N)	Mean difference (N)	p value	Max muscle activation (%)	Inter-subject SD (%)	Intra-subject SD (%)
TrapM.	I	36.55	74.36	57.51	12.05	–	–	18	5.36	1.18
II	17.00	101.01	62.06	26.44	4.54	N.S.D.	25	1.39	2.99
III	29.70	54.55	47.30	7.41	− 10.22	< 0.05	13	3.89	0.48
IV	13.69	66.21	43.51	17.43	− 14.01	< 0.05	16	2.76	0.52
TriM.	I	22.43	70.78	51.11	14.61	–	–	16	1.96	1.07
II	14.59	75.61	45.94	21.63	− 5.17	< 0.05	17	2.19	1.80
III	22.71	79.08	55.92	16.50	4.81	< 0.05	17	0.97	1.10
IV	.00	33.65	19.84	11.27	− 31.27	< 0.05	7	1.74	0.33
DeltM.	I	25.19	102.51	73.49	26.18	–	–	45	10.15	2.99
II	20.82	114.03	73.98	30.97	0.48	N.S.D.	50	11.70	3.26
III	83.87	162.59	134.24	24.34	60.75	< 0.05	71	6.75	3.33
IV	.00	52.60	22.95	21.14	− 50.54	< 0.05	23	12.46	1.50
SS	I	76.08	164.31	127.92	27.30	–	–	55	6.19	3.02
II	23.54	151.43	97.43	40.31	− 30.48	< 0.05	51	6.51	6.04
III	80.19	160.73	127.02	24.70	− 0.89	N.S.D.	54	9.58	3.16
IV	9.44	113.61	71.43	30.67	− 56.49	< 0.05	38	8.36	0.44
IS	I	59.73	161.28	117.50	33.64	–	–	27	7.34	1.86
II	18.96	143.15	79.29	39.07	− 38.21	< 0.05	24	3.66	3.88
III	77.97	221.55	160.93	42.39	43.43	< 0.05	37	5.12	1.39
IV	.00	91.81	52.36	34.45	− 65.14	< 0.05	15	3.88	0.86
BicL	I	25.71	44.11	37.30	5.80	–	–	15	6.18	1.65
II	13.84	50.83	33.49	11.85	− 3.80	< 0.05	17	6.75	2.74
III	52.86	99.58	80.63	15.06	43.33	< 0.05	34	9.45	1.35
IV	7.90	36.44	19.09	8.27	− 18.21	< 0.05	12	3.83	0.58

## References

[bb0010] Ackland D.C., Pandy M.G. (2009). Lines of action and stabilizing potential of the shoulder musculature. J. Anat..

[bb0005] Ackland D.C., Pak P., Richardson M., Pandy M.G. (2008). Moment arms of the muscles crossing the anatomical shoulder. J. Anat..

[bb0015] Bilal R.H. (2011). Rotator Cuff Pathology [Online]. Medscape.

[bb0020] Bryant D., Litchfield R., Sandow M., Gartsman G.M., Guyatt G., Kirkley A. (2005). A comparison of pain, strength, range of motion, and functional outcomes after hemiarthroplasty and total shoulder arthroplasty in patients with osteoarthritis of the shoulder: a systematic review and meta-analysis. J. Bone Joint Surg..

[bb0025] Cappozzo A., Catani F., Della Croce U., Leardini A. (1995). Position and orientation in-space of bones during movement—anatomical frame definition and determination. Clin. Biomech..

[bb0030] Charlton I.W., Johnson G. (2006). A model for the prediction of the forces at the glenohumeral joint. Proc. Inst. Mech. Eng. H J. Eng. Med..

[bb0035] data.gov.uk (2013). Driving Licence Data [Online]. http://data.dft.gov.uk/driving-licence-data/driving-licence-tables-nov2013.xls.

[bb0040] Haynes A. (2005). Design and evaluation of a device to test driving reaction time after shoulder surgery.

[bb0045] Kedgley A.E., Mackenzie G.A., Ferreira L.M., Drosdowech D.S., King G.J., Faber K.J., Johnson J.A. (2008). Humeral head translation decreases with muscle loading. J. Shoulder Elbow Surg..

[bb0050] Li Q., Xian C. (2013). Research of electric power steering system assistance characteristic based on the identification of the road. Adv. Mater. Res..

[bb0055] Lieber R.L., Friden J. (1993). Muscle damage is not a function of muscle force but active muscle strain. J. Appl. Physiol..

[bb0060] Ludewig P.M., Phadke V., Braman J.P., Hassett D.R., Cieminski C.J., LaPrade R.F. (2009). Motion of the shoulder complex during multiplanar humeral elevation. J. Bone Joint Surg..

[bb0065] Minigawa H., Itoi E. (2006). Clinical relevance of the rotator cuff in the shoulder with pain and dysfunction. Kansetsugelca.

[bb0070] OrthoLoad (2014). Database: Shoulder Joint – Turning Steering Wheel with Both Hands. [Online]. http://www.orthoload.com/?page_id=7.

[bb0075] Palmerud G., Forsman M., Sporrong H., Herberts P., Kadefors R. (2000). Intramuscular pressure of the infra- and supraspinatus muscles in relation to hand load and arm posture. Eur. J. Appl. Physiol..

[bb0080] Park S.J., Kim C.-B., Kim C.J., Lee J.W. (2000). Comfortable driving postures for Koreans. Int. J. Ind. Ergon..

[bb0085] Pick A.J., Cole D.J. (2006). Measurement of driver steering torque using electromyography. J. Dyn. Syst. Meas. Control..

[bb0090] Prinold J.A., Masjedi M., Johnson G.R., Bull A.M. (2013). Musculoskeletal shoulder models: a technical review and proposals for research foci. Proc. Inst. Mech. Eng. H J. Eng. Med..

[bb0095] Proske U., Morgan D.L. (2001). Muscle damage from eccentric exercise: mechanism, mechanical signs, adaptation and clinical applications. J. Physiol..

[bb0100] Rasmussen J., De Zee M. (2010). Year computational investigation of two interventions for neck and upper extremity pain in office workers. In 6th World Congress of Biomechanics (WCB 2010).

[bb0105] Rebiffe R. (1966). Paper 3: An Ergonomic Study of the Arrangement of the Driving Position in Motor Cars. Proceedings of the Institution of Mechanical Engineers, Conference Proceedings.

[bb0110] Smith C.D., Alexander S., Hill A.M., Huijsmans P.E., Bull A.M., Amis A.A., Beer J.F.D., Wallace A.L. (2006). A biomechanical comparison of single and double-row fixation in arthroscopic rotator cuff repair. J. Bone Joint Surg..

[bb0115] Solveig J., Johnsson B. (1975). Funtion of the muscles of the upper limb in car driving. Ergonomics.

[bb0120] Sporrong H., Palmerud G., Herberts P. (1996). Handgrip increases shoulder muscle activity, an EMG analysis with static hand contractions in 9 subjects. Acta Orthop. Scand..

[bb0125] Veeger H.E., van der Helm F.C. (2007). Shoulder function: the perfect compromise between mobility and stability. J. Biomech..

[bb0130] Westerhoff P., Graichen F., Bender A., Halder A., Beier A., Rohlmann A., Bergmann G. (2009). In vivo measurements of shoulder joint loads during activities of daily living. J. Biomech..

[bb0135] Wu G., van der Helm F.C.T., Veeger H.E.J., Makhsous M., Van Roy P., Anglin C., Nagels J., Karduna A.R., McQuade K., Wang X.G., Werner F.W., Buchholz B. (2005). ISB recommendation on definitions of joint coordinate systems of various joints for the reporting of human joint motion—Part II: shoulder, elbow, wrist and hand. J. Biomech..

[bb0140] Yamamoto A., Takagishi K., Osawa T., Yanagawa T., Nakajima D., Shitara H., Kobayashi T. (2010). Prevalence and risk factors of a rotator cuff tear in the general population. J. Shoulder Elbow Surg..

[bb0145] Yanagawa T., Goodwin C.J., Shelburne K.B., Giphart J.E., Torry M.R., Pandy M.G. (2008). Contributions of the individual muscles of the shoulder to glenohumeral joint stability during abduction. J. Biomech. Eng..

